# Diminished apoptosis in hypoxic porcine retina explant cultures through hypothermia

**DOI:** 10.1038/s41598-019-41113-4

**Published:** 2019-03-20

**Authors:** Ana M. Maliha, Sandra Kuehn, José Hurst, Fenja Herms, Michael Fehr, Karl U. Bartz-Schmidt, H. Burkhard Dick, Stephanie C. Joachim, Sven Schnichels

**Affiliations:** 1 Experimental Eye Research Institute, University Eye Hospital, Ruhr-University Bochum, Bochum, Germany; 2 University Eye Hospital Tübingen, Centre for Ophthalmology Tübingen, Tübingen, Germany; 3 Clinic for Small Animals, University of Veterinary Medicine, Hannover, Germany

## Abstract

Simulation of hypoxic processes *in vitro* can be achieved through cobalt chloride (CoCl_2_), which induces strong neurodegeneration. Hypoxia plays an important role in the progression of several retinal diseases. Thus, we investigated whether hypoxia can be reduced by hypothermia. Porcine retinal explants were cultivated for four and eight days and hypoxia was mimicked by adding 300 µM CoCl_2_ from day one to day three. Hypothermia treatment (30 °C) was applied simultaneously. Retinal ganglion, bipolar and amacrine cells, as well as microglia were evaluated via immunohistological and western blot analysis. Furthermore, quantitative real-time PCR was performed to analyze cellular stress and apoptosis. In addition, the expression of specific marker for the previously described cell types were investigated. A reduction of ROS and stress markers *HSP70*, *iNOS*, *HIF-1α* was achieved via hypothermia. In accordance, an inhibition of apoptotic proteins (*caspase 3*, *caspase 8*) and the cell cycle arrest gene *p21* was found in hypothermia treated retinae. Furthermore, neurons of the inner retina were protected by hypothermia. In this study, we demonstrate that hypothermia lowers hypoxic processes and cellular stress. Additionally, hypothermia inhibits apoptosis and protects neurons. Hence, this seems to be a promising treatment for retinal neurodegeneration.

## Introduction

A deprived oxygen supply in tissues is known as hypoxia and can occur in several retinal diseases, such as glaucoma^1^. A hallmark for hypoxic processes is the up-regulation of the transcription factor hypoxia inducible factor-1 (HIF-1), especially the stabilization of its oxygen sensitive subunit HIF-1α^2^. As a result, HIF-1α is translocated into the cell nucleus, where the expression of different hypoxic genes is induced^3,4^. Although cobalt is important for the neuronal integrity, high concentrations induce cytotoxic mechanisms by binding the oxygen-dependent region of HIF-1α and therefore prevent the degradation process of HIF-1α^5^. Furthermore, divalent metal ions, such as cobalt, can cause oxidative stress by rupturing the outer cell membrane and disturbing the mitochondrial respiration. These mechanisms of cellular toxicity have been proposed for several neurodegenerative disorders. Through its characteristics as a hypoxia mimicking agent, cobalt chloride is commonly used for the induction of neurodegeneration in different models^6–10^. In a previous study, we evaluated the effects of different CoCl_2_ concentrations on porcine retinae and demonstrated that it induced neuronal cell loss, which was associated with increased apoptosis mechanisms^11^. Further previous performed studies, which evaluated the effect of hypoxia induced by oxygen (O_2_)-deprivation, point out that always a change of the same parameters in both models was observed^12–15^. Therefore, hypoxia via CoCl_2_ is to some extent comparable to hypoxia induced by O_2_-depriviation.

Hypothermia, described as temperature below 37 °C, seems to have neuroprotective effects, although the underlying molecular mechanism is not completely understood yet^16,17^. Nevertheless, several neuroprotective effects of hypothermia on the retina were reported. Rat retinae were protected from ischemia/reperfusion induced damage by hypothermia^18^. Bovine retinal ganglion cells (RGCs) showed prolonged survival under ischemic conditions after hypothermia and RGCs from minipigs were protected from ischemia induced cell loss^12,14^.

The goal of our study was to investigate possible neuroprotective effects of hypothermia in a CoCl_2_ induced degeneration model of cultured porcine retinal explants. Hence, hypothermia at 30 °C was applied to retinal explants and hypoxic processes and cellular stress markers were evaluated. Furthermore, the apoptotic conditions of whole retinae and the apoptosis rate of RGCs were analyzed. In addition, bipolar and amacrine cells as well as glial cells were assessed after four and eight days of cultivation.

Here, we prove that hypothermia has neuroprotective effects on CoCl_2_ treated retinae by reducing hypoxic processes, cellular stress and inhibiting apoptosis. In conclusion, a rescue of neurons, especially RGCs, was achieved.

## Results

**Figure 1:**
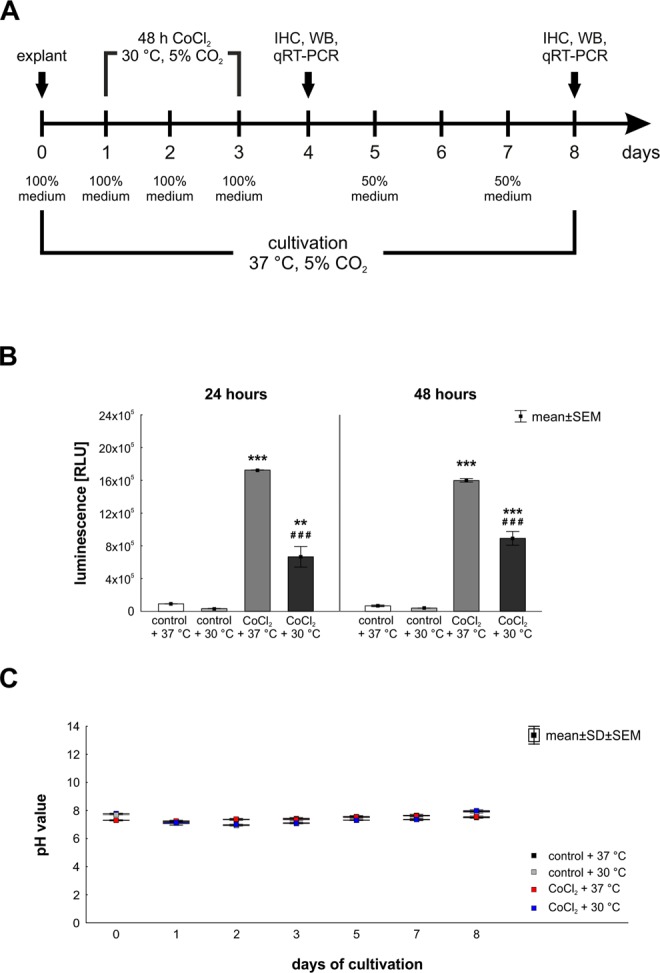
(**A**) Study timeline. Explants of porcine retinae were prepared at day zero and cultivated for four and eight days. Degeneration processes were induced by adding CoCl_2_ (300 µM) from day one to day three. Hypothermia treatment (30 °C) was applied simultaneously. Four groups were compared: control + 37 °C, CoCl_2_ + 37 °C, hypothermia treated control + 30 °C and CoCl_2_ + 30 °C. At days four and eight retina samples were prepared for immunohistological (IHC), western blot (WB) and qPCR analyses. (**B**) Hypothermia reduced the ROS-production in cultivated retina. ROS-level was measured 24 and 48 hours after CoCl_2_-induction. For both points in time, the ROS-level was strongly elevated after CoCl_2_-treatment. Hypothermia reduced the ROS-production significantly in CoCl_2_-treated retinae. However, it was still higher than in control + 37 °C retinae. (**C**) pH-value was measured to assure that degenerative effects were induced by CoCl_2_ and not by cultivation effects. pH-value was stable at any day of cultivation. B: n = 3/group. C: n = 10/group. **p < 0.01; ^###^,***p < 0.001.

Hypothermia treatment (30 °C) and hypoxia (300 µM CoCl_2_) were performed simultaneously (Fig. 1A). After four and eight days, retinal explants were obtained for quantitative real-time PCR (qPCR), histological and western blot analyses. Additionally, pH-measurements were performed after each medium exchange, and reactive oxygen species (ROS) level was evaluated on days one and two.

To assure that degenerative effects were induced by CoCl_2_, we analyzed the oxidative stress by measuring the level of ROS in cultured retinae 24 and 48 hours after CoCl_2_-induction (Fig. 1B). In both investigated points in time, the ROS-level of CoCl_2_ + 37 °C treated retinae (24 h: 1,725,425 ± 3,073.1 RLU; p = 0.0002; 48 h: 1,597,542 ± 18,806.7 RLU; p = 0.0002) was strongly elevated in comparison to control + 37 °C retinae (24 h: 91,389 ± 3,117.6 RLU; 48 h: 67,404 ± 1,008.2 RLU). Interestingly, hypothermia reduced the ROS-level in CoCl_2_ + 30 °C treated retinae (24 h: 666,153 ± 125,548.3 RLU; 48 h: 891,382 ± 83,562.2 RLU) significantly in contrast to CoCl_2_ + 37 °C retinae (for both: p = 0.0002; Fig. 1B). Additionally, we measured the pH-value of the media after each medium change. No differences were seen within the groups for each point in time, indicating that degenerative effects were not induced by the cultivation of retinae (Fig. 1C).

For further investigations of the effects of hypothermia on CoCl_2_, we performed hematoxylin & eosin staining of retinal cross-sections (Sup. Fig. S1A). As described previously^11^, CoCl_2_ lead to a reduction of the retinal thickness. To evaluate whether hypothermia inhibited neurodegenerative effects of CoCl_2_ on porcine retina, retinal thickness was measured. At both investigated points in time, the retinal thickness was reduced significantly through CoCl_2_ (4 days: p = 0.01; 8 days: 0.03) in comparison to control retinae. For both points in time, four and eight days, hypothermia preserved retinal thickness, that CoCl_2_ + 30 °C treated retinae were significantly thicker than CoCl_2_ + 37 °C treated ones (4 days: p = 0.003; 8 days: p = 0.002) and no difference were seen between CoCl_2_ + 30 °C retinae and control + 37 °C retinae (p > 0.6; Sup. Fig. S1B). These results indicate that hypothermia lowers oxidative stress induced by CoCl_2_, and preserved retinal thickness, which was reduced by CoCl_2_-treatment.

### Hypothermia inhibited hypoxic processes and reduced cellular stress

**Figure 2:**
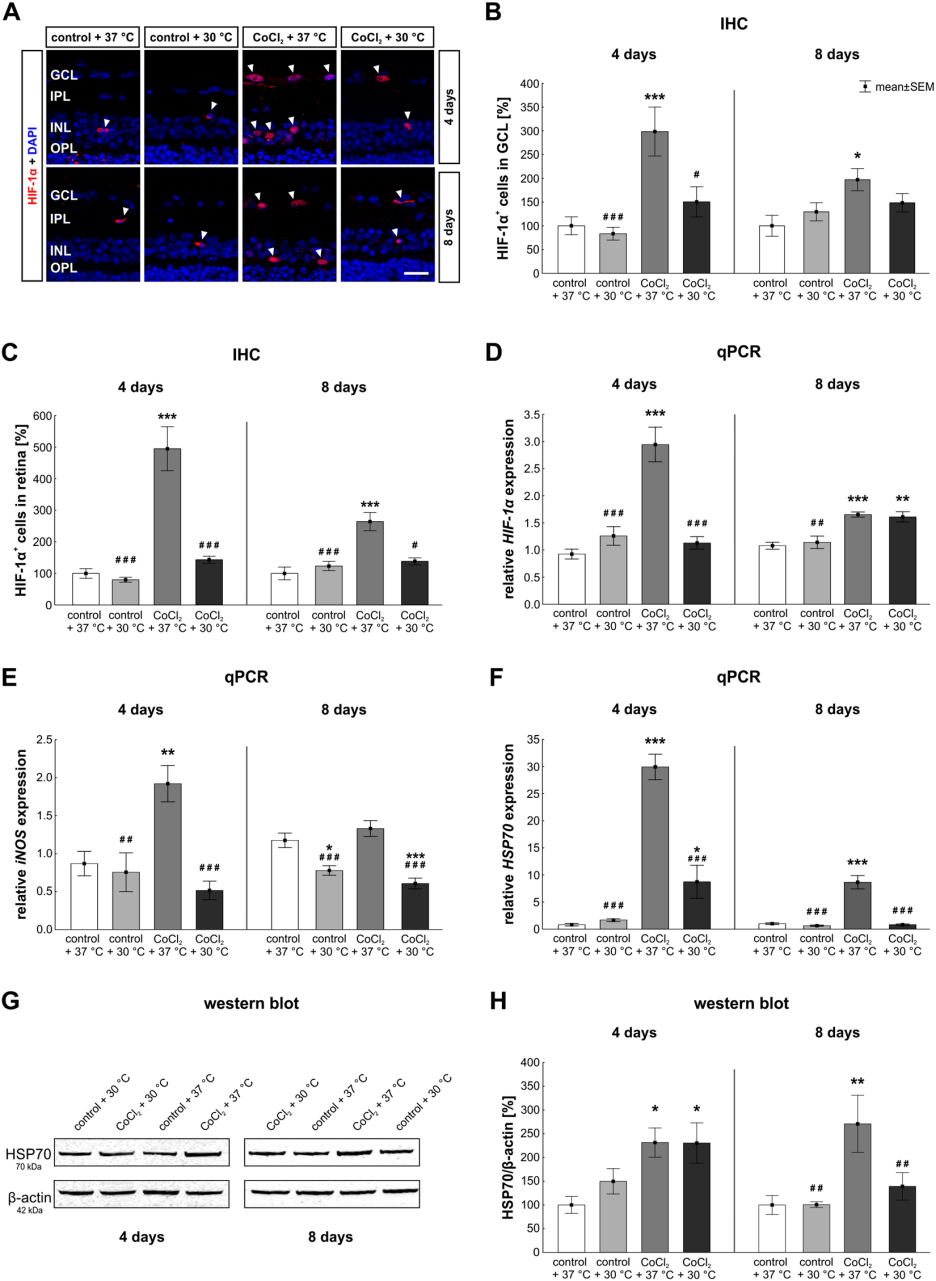
Reduced hypoxic processes and cellular stress through hypothermia. (**A**) Representative pictures of hypoxic cells in retinae. Hypoxic cells were stained with anti-HIF-1α (red, arrowheads). DAPI was used to visualize cell nuclei (blue). (**B**) Statistical evaluation of HIF-1α cell counts showed, that CoCl_2_ led to a strongly elevated number of HIF-1α^+^ cells located in the GCL after four and eight days. Hypothermia inhibited hypoxic processes and lowered hypoxia in most of the cells located in the GCL. (**C**) In regard to the number of hypoxic cells in the whole retina, CoCl_2_ again led to an increased hypoxia, whereas hypothermia alleviated hypoxic processes. (**D**) mRNA levels of *HIF-1α* were evaluated via qPCR. Analyses revealed an increased *HIF-1α* mRNA expression in the CoCl_2_ + 37 °C group at both days compared to the control + 37 °C group. At day four, hypothermia led to a control-like *HIF-1α* expression. (**E**) mRNA levels of *iNOS* were analyzed with qPCR. The *iNOS* mRNA expression was significantly increased by CoCl_2_ after four days. This effect was counteracted by hypothermia. At day eight, CoCl_2_ had no effect on *iNOS* expression, whereas both hypothermia treated groups, showed a reduced *iNOS* mRNA expression in comparison to the control group. (**F**) qPCR analysis regarding *HSP70*. CoCl_2_ strongly elevated the *HSP70* mRNA expression level at days four and eight. At both points in time, hypothermia lowered *HSP70* mRNA expression in the CoCl_2_ stressed retinae. (**G**) Protein levels of HSP70 (70 kDa) were measured via western blot and normalized against β-actin (42 kDa). (**H**) At day four, a significantly increased signal intensity of HSP70 was noted in both CoCl_2_ treated group, irrespectively of the temperature. Hypothermia treatment decreased the HSP70 signal intensity after eight days, causing no difference between the CoCl_2_ + 30 °C and the control + 37 °C group. Abbreviations: GCL = ganglion cell layer; IPL = inner plexiform layer; INL = inner nuclear layer; OPL = outer plexiform layer; IHC = immunohistochemistry; qPCR = quantitative real-time PCR. Values are mean ± SEM. B, C: n = 9–10/group; D-H: n = 6–7/group. Statistical differences to control + 37 °C group are marked with * and differences to CoCl_2_ + 37 °C group with ^#^. ^#^,*p < 0.05; ^##^,**p < 0.01; ^###^,***p < 0.001. Scale bar = 20 µm.

Since the accumulation of the transcription factor HIF-1α is a hallmark for hypoxic conditions^7^, hypoxic cells were stained with anti-HIF-1α antibody (Fig. 2A). To investigate whether CoCl_2_-treatment indeed induced hypoxic processes in retinae, the amount of HIF-1α^+^ cells located in the ganglion cell layer (GCL, Fig. 2B) as well as in the whole retina (Fig. 2C) was evaluated at four and eight days. At four days, CoCl_2_ + 37 °C treated retinae showed three times as many HIF-1α^+^ cells in the GCL (298.4 ± 51.6% HIF-1α^+^ cells/GCL; p = 0.0009) and even five times more in the whole retina (494.9 ± 69.8% HIF-1α^+^ cells/retina; p = 0.0002) in comparison to the control ones (100.0 ± 19.0% HIF-1α^+^ cells/GCL; 100.0 ± 15.3% HIF-1α^+^ cells/retina). Hypothermia treatment led to a significantly reduced amount of HIF-1α^+^ cells in the GCL (150.4 ± 31.8% HIF-1α^+^ cells/GCL; p = 0.015) as well as in the whole retina (143.4 ± 10.9% HIF-1α^+^ cells/retina; p = 0.0002) when compared to CoCl_2_ + 37 °C retinae. No statistical differences were seen between control groups and hypothermia treated CoCl_2_ + 30 °C groups.

After eight days, CoCl_2_ led to a significant increase of HIF-1α^+^ cells in the GCL (197.1 ± 23.4% HIF-1α^+^ cells/GCL; p = 0.013) and in the whole retina (264.1 ± 28.8% HIF-1α^+^ cells/retina; p = 0.0002) in comparison to control retinae (100.0 ± 22.1% HIF-1α^+^ cells/GCL; 100.0 ± 20.2% HIF-1α^+^ cells/retina). Interestingly, after hypothermia treatment the amount of HIF-1a^+^ cells was reduced in the GCL (148.5 ± 19.5% HIF-1α^+^ cells/GCL; p = 0.42) and significantly decreased in the whole retina (138.5 ± 11.0% HIF-1α^+^ cells/retina; p = 0.001) to the extent, that no statistical difference was seen between control retinae and hypothermia treated CoCl_2_ + 30 °C retinae (GCL: p = 0.42; retina: p = 0.58; Fig. 2B,C). To verify that CoCl_2_ indeed induces hypoxia and hypothermia lowers the number of hypoxic cells, qPCR analyses of the *HIF-1α* expression level were performed (Fig. 2D). At day four, the *HIF-1α* expression was significantly increased after CoCl_2_ induction (2.9 ± 0.8-fold; p = 0.0002) compared to control + 37 °C retinae. As seen in the cell counting, hypothermia treatment reduced the *HIF-1α* mRNA expression in the CoCl_2_ + 30 °C group (1.1 ± 0.3-fold; p = 0.0002) compared to the CoCl_2_ + 37 °C group. Most importantly, no differences between the control + 37 °C and the CoCl_2_-stressed hypothermia group (p = 0.89) were noted (Fig. 2D), indicating a complete counteraction of the stressor via hypothermia. After eight days, the *HIF-1α* expression was more prominent in the CoCl_2_ + 37 °C group (1.65 ± 013-fold) than in the control group (37 °C: 1.1 ± 0.2-fold; p = 0.0007). In contrast to the results at day four and the cell counts, hypothermia treatment had no inhibiting effect on the *HIF-1α* expression after eight days, as no difference between the CoCl_2_ + 30 °C (1.6 ± 0.3-fold; p > 0.9) and the CoCl_2_ + 37 °C group was notable (Fig. 2D).

For further investigations on cellular stress, the *iNOS* mRNA levels in retinae were analyzed (Fig. 2E). After four days of cultivation, a 1.9 ± 0.6-fold higher *iNOS* expression was noted in CoCl_2_-stressed retinae compared to control + 37 °C retinae (p = 0.007). However, hypothermia counteracted the effect of CoCl_2_ and significantly reduced the *iNOS* expression (0.5 ± 0.3-fold; p = 0.0002) in comparison to CoCl_2_ + 37 °C. Again, no differences were notable between hypothermia treated CoCl_2_-stressed retinae and control ones (p = 0.612; Fig. 2E). After eight days, hypothermia (0.8 ± 0.2-fold; p = 0.015) caused a significantly decreased *iNOS* expression in comparison to the control + 37 °C group. Interestingly, no alterations were seen comparing CoCl_2_-stressed retinae (p = 0.570) and control ones. In contrast, a significantly reduced mRNA expression of *iNOS* was found in the CoCl_2_ + 30 °C group (0.6 ± 0.2-fold; p = 0.0006) compared to the CoCl_2_ + 37 °C group (Fig. 2E).

HSP70, a chaperon belonging to the heat shock protein family, is important for the correct folding process of proteins, and accumulates under stress conditions^19^. To evaluate the effect of CoCl_2_ and hypothermia on cellular stress *HSP70* mRNA expression was analyzed (Fig. 2F). At the early point in time, the *HSP70* expression level was significantly higher in the CoCl_2_ + 37 °C group (29.8 ± 5.7-fold; p = 0.0002) than in the control + 37 °C. CoCl_2_-stressed retinae treated with hypothermia (8.7 ± 7.4-fold; p = 0.04) still had an increased *HSP70* expression compared to control ones, but interestingly, lowering the temperature significantly diminished the *HSP70* expression in comparison to the CoCl_2_-stressed retinae at 37 °C (p = 0.0002). After eight days, the CoCl_2_ + 37 °C group (8.6 ± 3.2-fold; p = 0.0002) presented a significantly increased mRNA expression level compared to the control + 37 °C group. Hypothermia treatment led to a significant reduction of *HSP70* mRNA expression in the CoCl_2_-stressed hypothermia group (0.8 ± 0.1-fold; p = 0.0002) in comparison to the CoCl_2_ + 37 °C group. Most importantly, these results prove the complete inhibition of cellular stress after hypothermia treatment since no differences were seen between the CoCl_2_ + 30 °C group and the control + 37 °C group (p > 0.9; Fig. 2F). Additionally, we performed western blot analyses to evaluate HSP70 protein levels (Fig. 2G,H). A significantly increased signal intensity of HSP70 was noted after four days in the CoCl_2_ + 37 °C group (231.4 ± 30.8%) in comparison to the control + 37 °C group (100.0 ± 17.8%; p = 0.015). In contrast to the results of qPCR analyses regarding HSP70, the signal intensity of HSP70 was increased in the CoCl_2_ + 30 °C group (230.5 ± 42.5%) in comparison to the control group (p = 0.044). Nevertheless, western blot analyses of HSP70 after eight days, were in accordance with those results seen in the qPCR. The addition of CoCl_2_, at 37 °C, led to a strongly increased signal intensity (271.0 ± 60.3%) in comparison to the control + 37 °C group (100.0 ± 19.9%; p = 0.005). Interestingly, hypothermia treatment normalized the signal intensity of HSP70 completely, resulting in no differences between the CoCl_2_ + 30 °C (139.3 ± 29.1%) and the control + 37 °C group (p = 0.79; Fig. 2H). In summary, these findings indicate a total counteraction of cellular stress and an early prevention of hypoxic processes in CoCl_2_-stressed retinae after hypothermia treatment.

### Neuroprotection via hypothermia

RGCs transfer the electrochemical information via their axons, which build the optic nerve, to the brain. Since RCGs are affected in glaucoma it is important to establish new therapeutic approaches that protect retinal neurons, most of all RGCs.

**Figure 3:**
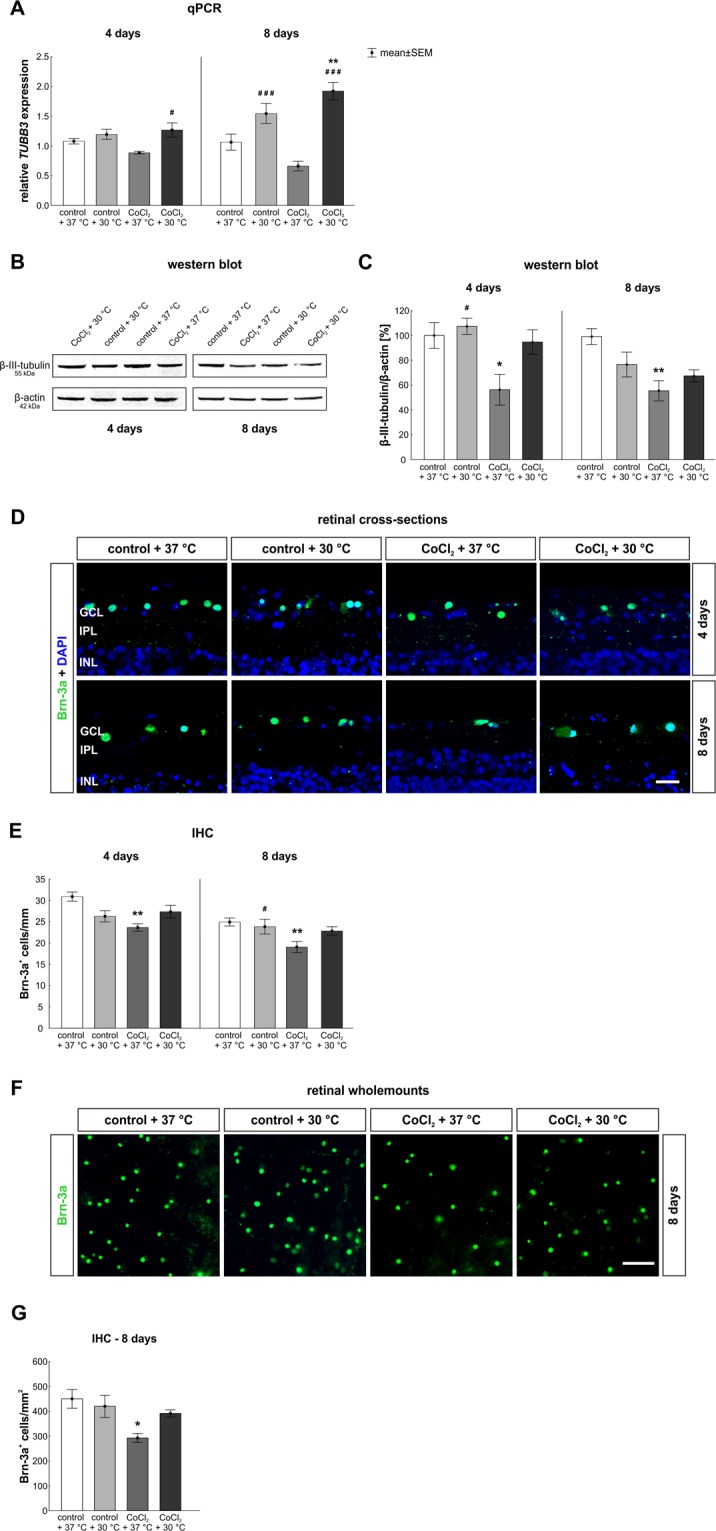
Protection of neurons, especially of retinal ganglion cells (RGCs), after hypothermia. (**A**) qPCR analysis regarding *TUBB3*. At day eight, a higher *TUBB3* mRNA expression was found in both hypothermia treated groups. (**B**) Protein levels of β-III-tubulin, at 55 kDA, were measured via western blot and normalized against β-actin, at 42 kDa. (**C**) A significantly reduced β-III-tubulin protein level was observed after four and eight days via western blot analyses in the CoCl_2_ + 37 °C group. This effect was counteracted by hypothermia treatment. (**D**) Representative pictures of the ganglion cell layer. RGCs were stained in retinal cross-sections with anti-Brn-3a (green) at days four and eight. Cell nuclei were labelled with DAPI (blue). (**E**) Quantification revealed that CoCl_2_ at 37 °C induced a RGC loss after four and eight days. Degenerative effects of CoCl_2_ were counteracted via hypothermia treatment at both points in time. (**F**) Representative images depict RGCs stained in wholemount retinae at eight days using anti-Brn-3a (green). (**G**) Also in wholemounts, a significant loss was noted in the CoCl_2_ treated retinae at 37 °C, whereas hypothermia treatment protected RCGs. Abbreviations: GCL = ganglion cell layer; IPL = inner plexiform layer; INL = inner nuclear layer. qPCR = quantitative real-time PCR; IHC = Immunohistochemistry. Values are mean ± SEM. A: n = 6–7/group, B,C : n = 4/group; E,G: n = 10/group. Statistical differences to control + 37 °C group are marked with * and differences to CoCl_2_ + 37 °C group with ^#^. ^#^,*p < 0.05, **p < 0.01, ^###^p < 0.001. Scale bar = 20 µm (**D**); scale bar = 50 µm (**F**).

To evaluate the possible neuroprotective effects of hypothermia on RGCs, we measured the *TUBB3* expression level via qPCR analysis (Fig. 3A). At the early point in time, both, the hypothermia alone (1.2 ± 0.2-fold; p = 0.73) and the CoCl_2_ treated retinae at 37 °C (0.9 ± 0.1-fold; p = 0.32), had a similar *TUBB3* expression as control retinae. A 1.3 ± 0.3-fold increased *TUBB3* expression level was noted in hypothermia treated CoCl_2_-stressed retinae (p = 0.013) compared to CoCl_2_ stressed retinae at 37 °C (Fig. 3A). At day eight, the *TUBB3* level in the CoCl_2_-stressed retinae (0.66 ± 0.2-fold; p = 0.189) was not altered in comparison to the control + 37 °C. In contrast, hypothermia treatment increased the *TUBB3* expression in the CoCl_2_ + 30 °C retinae (1.9 ± 0.4-fold; p = 0.001) compared to control and CoCl_2_-stressed retinae at 37 °C (p = 0.0002; Fig. 3A).

In addition, the β-III-tubulin protein level was investigated via western blot (Fig. 3B,C). At day four, CoCl_2_ + 37 °C treated retinae (56.3 ± 12.4%; p = 0.041) presented a significantly diminished signal intensity in comparison to the control + 37 °C (100.0 ± 10.4%). Hypothermia counteracted the harmful effects of CoCl_2_ on β-III-tubulin and led to a 1.69-fold increased protein level in the CoCl_2_ + 30 °C group (94.7 ± 9.8%; p = 0.078) in comparison to the CoCl_2_ + 37 °C group. In accordance with prior results, neuroprotective effects of hypothermia were noted since no differences in the β-III-tubulin intensity were found after hypothermia treatment between the control + 37 °C and CoCl_2_ + 30 °C group (p > 0.9; Fig. 3C). After eight days, the CoCl_2_ + 37 °C retinae (55.9 ± 8.2%; p = 0.007) still had an attenuated protein level in contrast to the control + 37 °C ones (100.0 ± 6.4%). Hypothermia diminished the CoCl_2_ effects on neurons, but nevertheless a slight reduction was seen in the CoCl_2_ + 30 °C retinae (68.0 ± 4.9%; p = 0.051) compared to the control + 37 °C retinae (Fig. 3C).

For histological evaluation of RGCs, an anti-Brn-3a antibody was used for the staining on retinal cross-sections and retinal wholemounts (Fig. 3D,F). After four days, significantly fewer Brn-3a^+^ cells were detected in cross-sections of CoCl_2_ + 37 °C treated retinae (23.7 ± 0.9 Brn-3a^+^ cells/mm; p = 0.001) than in control retinae (30.9 ± 1.1 Brn-3a^+^ cells/mm). Some protection of Brn-3a^+^ RGCs through hypothermia was achieved in the CoCl_2_ + 30 °C group (27.4 ± 1.5 Brn-3a^+^ cells; p = 0.158) in comparison to the CoCl_2_ + 37 °C group (Fig. 3E). At day eight, a significant RGC loss in the CoCl_2_ + 37 °C group (18.8 ± 1.2 Brn-3a^+^ cells/mm; p = 0.003) was prevented by hypothermia treatment (CoCl_2_ + 30 °C: 22.4 ± 0.8 Brn-3a^+^ cells/mm). In comparison to the CoCl_2_ + 37 °C group, RGCs were slightly preserved after hypothermia treatment (p = 0.155) and no differences were observed between control + 37 °C (25.1 ± 0.9 Brn-3a^+^ cells/mm) and CoCl_2_ + 30 °C groups (p = 0.394; Fig. 3E). To confirm these findings, RGCs were also stained on wholemount retinae (Fig. 3F). Results in wholemounts were in accordance with those seen in retinal cross-sections. A significant loss of RGCs was noted in the CoCl_2_ + 37 °C group (292.9 ± 18.2 Brn-3a^+^ cells/mm^2^; p = 0.031) when compared to control + 37 °C retinae (450.4 ± 38.2 Brn-3a^+^ cells/mm^2^; Fig. 3G). Protection of RGCs due to hypothermia were also seen in whole mount retinae, since CoCl_2_ + 30 °C retinae (391.8 ± 14.5 Brn-3a^+^ cells/mm^2^; p > 0.9) had a similar number of Brn-3a^+^ RGCs as control + 37 °C retinae. The protection via hypothermia was so prominent, that the amount of RGCs in hypothermia treated retinae tended to be higher in CoCl_2_ + 30 °C retinae than in CoCl_2_ + 37 °C ones (p = 0.198; Fig. 3G). With these results, we can state that hypothermia completely counteracted the harming effects of CoCl_2_ on RGCs.

### Partial protection of the inner nuclear layer

**Figure 4:**
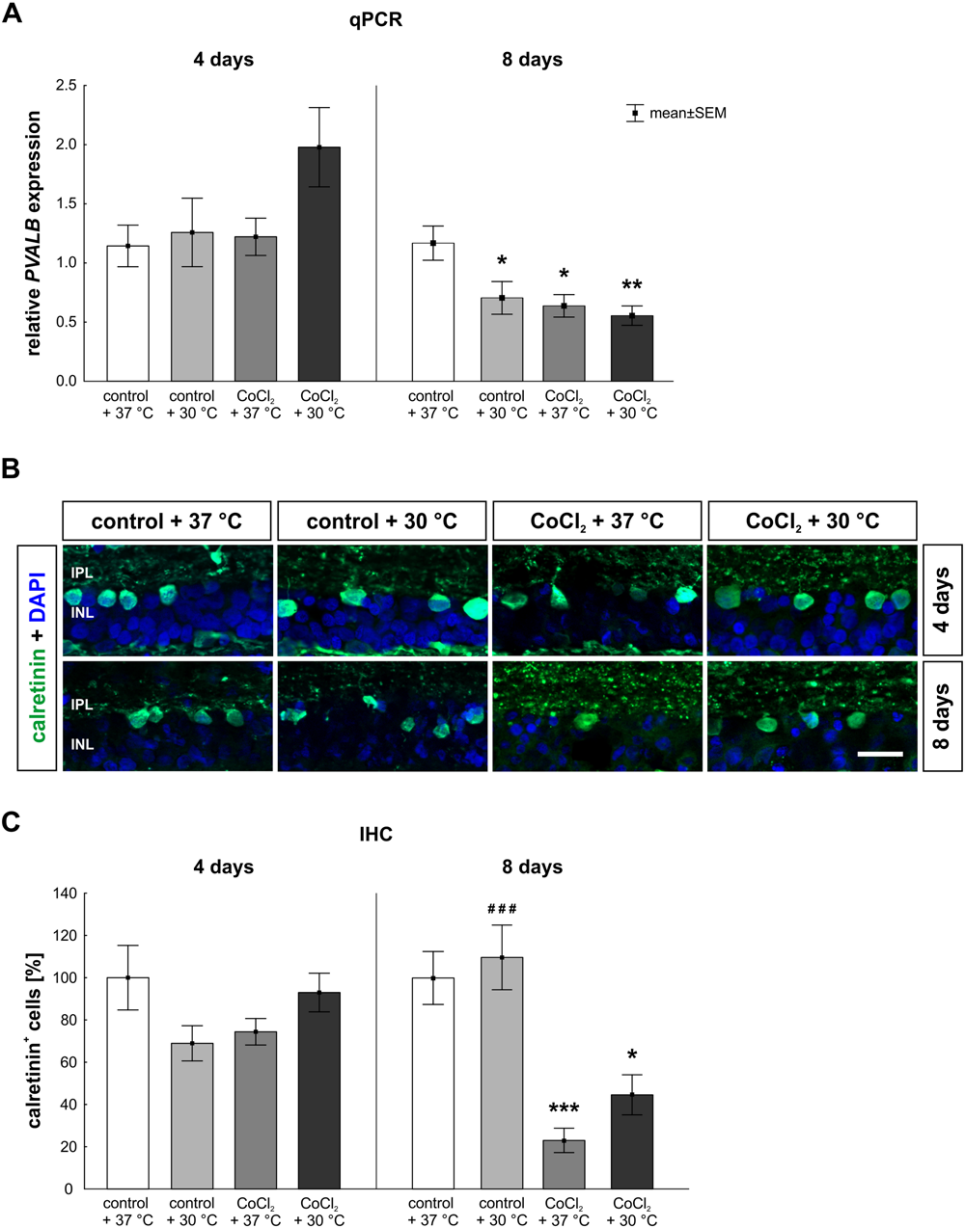
Late loss of amacrine cells. (**A**) qPCR analysis of *PVALB*. After eight days, a significantly reduced mRNA expression of *PVALB* was observed in all groups when compared to the control + 37 °C. (**B**) Representative pictures of the inner layers. Amacrine cells were stained with anti-calretinin (green) at four and eight days of cultivation. DAPI was used to visualize the cell nuclei (blue). (**C**) At day eight, a significant loss of amacrine cells was detected in the CoCl_2_ + 37 °C group. Hypothermia treatment did not rescue the amacrine cells. Abbreviations: IPL = inner plexiform layer; INL = inner nuclear layer; qPCR = quantitative real-time PCR; IHC = immune-histochemistry. Values are mean ± SEM. A: n = 6–7/group, B, C: n = 9–10/group. Statistical differences to control + 37 °C group are marked with * and differences to CoCl_2_ + 37 °C group with ^#^. *p < 0.05; **p < 0.01; ^###^,***p < 0.001. Scale bar = 20 µm.

**Figure 5:**
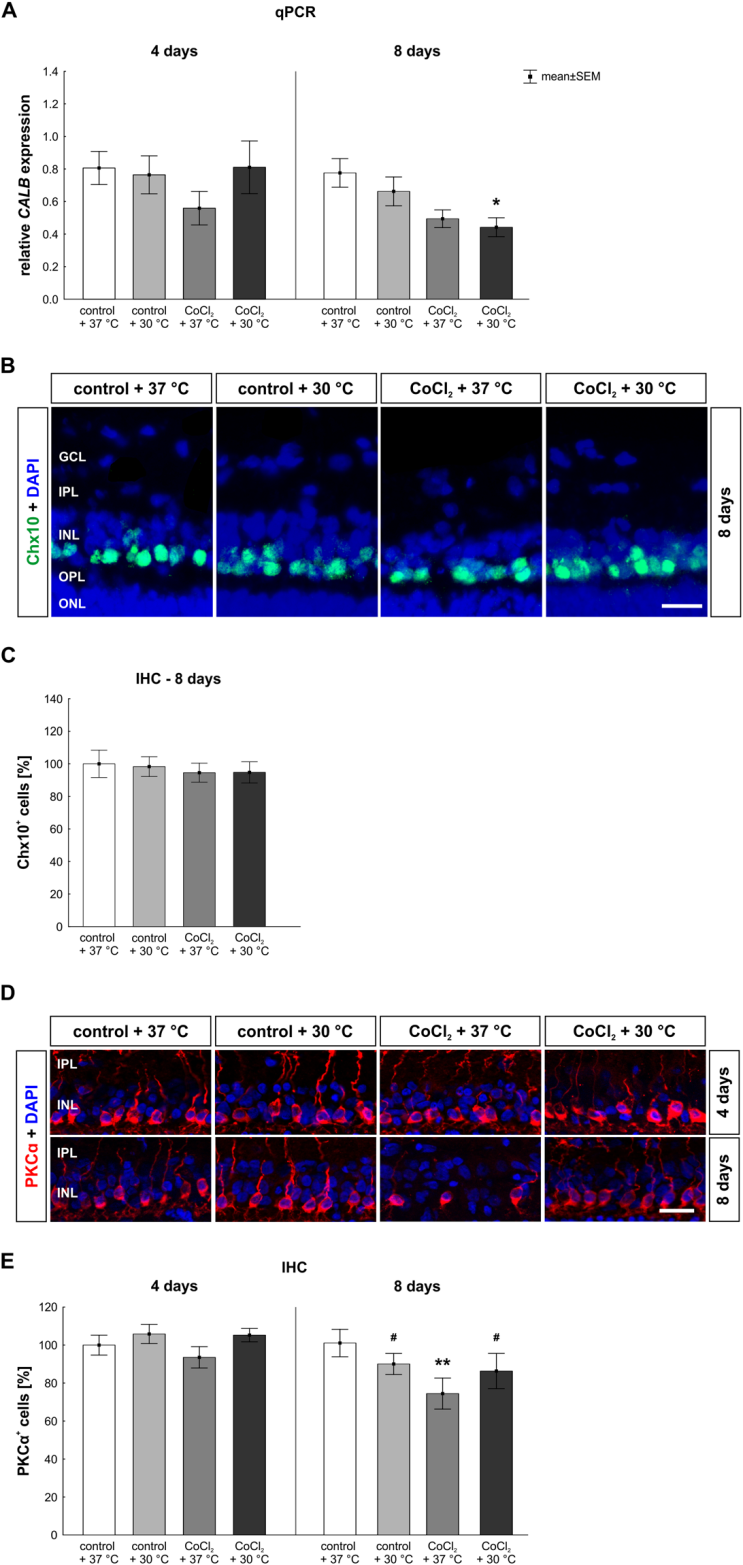
Late loss of bipolar cells was counteracted by hypothermia. (**A**) *CALB* mRNA expression was measured via qPCR. After eight days, a significantly decreased *CALB* mRNA expression was seen in the hypothermia treated CoCl_2_ + 30 °C retinae. (**B**) Representative images of bipolar cells stained with anti-Chx10 (green) at eight days. DAPI was used for the visualization of cell nuclei (blue). (**C**) After eight days, all groups had a similar number of Chx10^+^ cells. (**D**) Representative pictures of the inner layers are given. Rod bipolar cells (red) were stained immunohistochemically at days four and eight using an anti-PKCα antibody. Cell nuclei are shown in blue. (**E**) At day eight, a significant loss of bipolar cells was noted in the CoCl_2_ + 37 °C. A rescue of PKCα^+^ cells was achieved by hypothermia. Abbreviations: GCL = ganglion cell layer; IPL = inner plexiform layer; INL = inner nuclear layer; OPL = outer plexiform layer; ONL = outer nuclear layer; qPCR = quantitative real-time PCR; IHC = immunohistochemistry. Values are mean ± SEM. A: n = 6–7/group; C,E: n = 9–10/group. Statistical differences to control + 37 °C group are marked with * and differences to CoCl_2_ + 37 °C group with ^#^. ^#^,*p < 0.05; **p < 0.01 Scale bar = 20 µm.

Based on the previous findings that hypothermia lowers cellular stress and protects RGCs, we were interested if hypothermia also has protective effects on other retinal cell types. To this end, we investigated amacrine (Fig. 4) and bipolar cells (Fig. 5) which are both located in the inner nuclear layer.

At day four, hypothermia treatment led to a slightly increased *PVALB* mRNA expression, a gene expressed by amacrine cells (CoCl_2_ + 30 °C: 2.0 ± 0.8-fold; p = 0.119; Fig. 4A), whereas the other groups presented an unaltered expression compared to the control retinae. At day eight, all groups (control + 30 °C: 0.7 ± 0.4-fold; p = 0.048; CoCl_2_ + 37 °C: 0.6 ± 0.3-fold; p = 0.019; CoCl_2_ + 30 °C: 0.6 ± 0.2-fold; p = 0.006) had a lower *PVALB* expression than the control + 37 °C group (Fig. 4A).

In histological analyses, the number of calretinin labeled amacrine cells was evaluated (Fig. 4B,C). Neither CoCl_2_ (37 °C: 74.4 ± 6.3% calretinin^+^ cells; p = 0.311; 30 °C: 93.0 ± 9.1% calretinin^+^ cells; p > 0.9) nor hypothermia alone (68.9 ± 8.33% calretinin^+^ cells; p = 0.163) had any impact on the amount of calretinin^+^ cells compared to control + 37 °C retinae (100.0 ± 15.3% calretinin^+^ cells; Fig. 4C). In contrast, after eight days, in retinae treated with CoCl_2_, irrespectively of the temperature (37 °C: 24.3 ± 6.1% calretinin^+^ cells; p = 0.0005; 30 °C: 47.0 ± 10.0% calretinin^+^ cells; p = 0.016), significant fewer amacrine cells were counted than in control + 37 °C retinae (100.0 ± 12.5% calretinin^+^ cells; Fig. 4C).

The mRNA expression of *calbindin* (*CALB*), a gene expressed by horizontal cells, was not altered in any of the groups compared (CoCl_2_ + 37 °C: 0.5 ± 0.3-fold; CoCl_2_ + 30 °C: 0.8 ± 0.4-fold; p > 0.5) to the control + 37 °C group at day four (Fig. 5A). Only at day eight, a slight reduction was observed in the CoCl_2_ + 37 °C group (0.5 ± 0.1-fold; p = 0.058) and even a significant reduction of the *CALB* expression level was noted in the CoCl_2_ + 30 °C group (0.4 ± 0.2-fold; p = 0.019) in comparison to the control (Fig. 5A).

For investigations of the bipolar cell-population we started with a pan-bipolar cell marker anti-Chx10 after eight days (Fig. 5B). The number of Chx10^+^ bipolar cells was not altered in any of the groups (control + 30 °C: 98.31 ± 6.08% Chx10^+^ cells; CoCl_2_ + 37 °C: 94.57 ± 5.85% Chx10^+^ cells; CoCl_2_ + 30 °C: 94.76 ± 6.52% Chx10^+^ cells) in comparison to the control + 37 °C (100.00 ± 8.40% Chx10^+^ cells; for all: p > 0.9; Fig. 5C).

Since it is known that CoCl_2_ leads to a degeneration of rod bipolar cells^11^, we additionally investigated the amount of PKCα^+^ rod bipolar cells after four and eight days (Fig. 5D,E). Regarding the amount of PKCα^+^ rod bipolar cells, the number of bipolar cells was not changed in any of the groups in comparison to the control + 37 °C group (100.0 ± 5.2% PKCα^+^ cells; Fig. 5E) at day four. However, at day eight, a significant loss of bipolar cells was noted in the CoCl_2_ + 37 °C group (65.7 ± 5.3% PKCα^+^ cells; p = 0.006) in comparison to control + 37 °C retinae (100.0 ± 8.0% PKCα^+^ cells). Even more, a total rescue of PKCα^+^ cells was achieved by hypothermia (CoCl_2_ + 30 °C: 94.1 ± 7.1% PKCα^+^ cells; p = 0.029), resulting in no difference between control + 37 °C and hypothermia treated CoCl_2_ + 30 °C retinae (p > 0.9; Fig. 5E).

These findings show, that the damaging effect of CoCl_2_ on amacrine cells was not attenuated by hypothermia. While the total bipolar cell population was not affected neither by CoCl_2_ nor by hypothermia, the late loss of rod-bipolar cells was totally counteracted after hypothermia.

### Apoptotic mechanisms were reduced through hypothermia

To investigate underlying mechanisms that lead to protection of RGCs after hypothermia, we evaluated apoptosis. To this end, we analyzed the expression of several genes that are involved in apoptosis.

**Figure 6:**
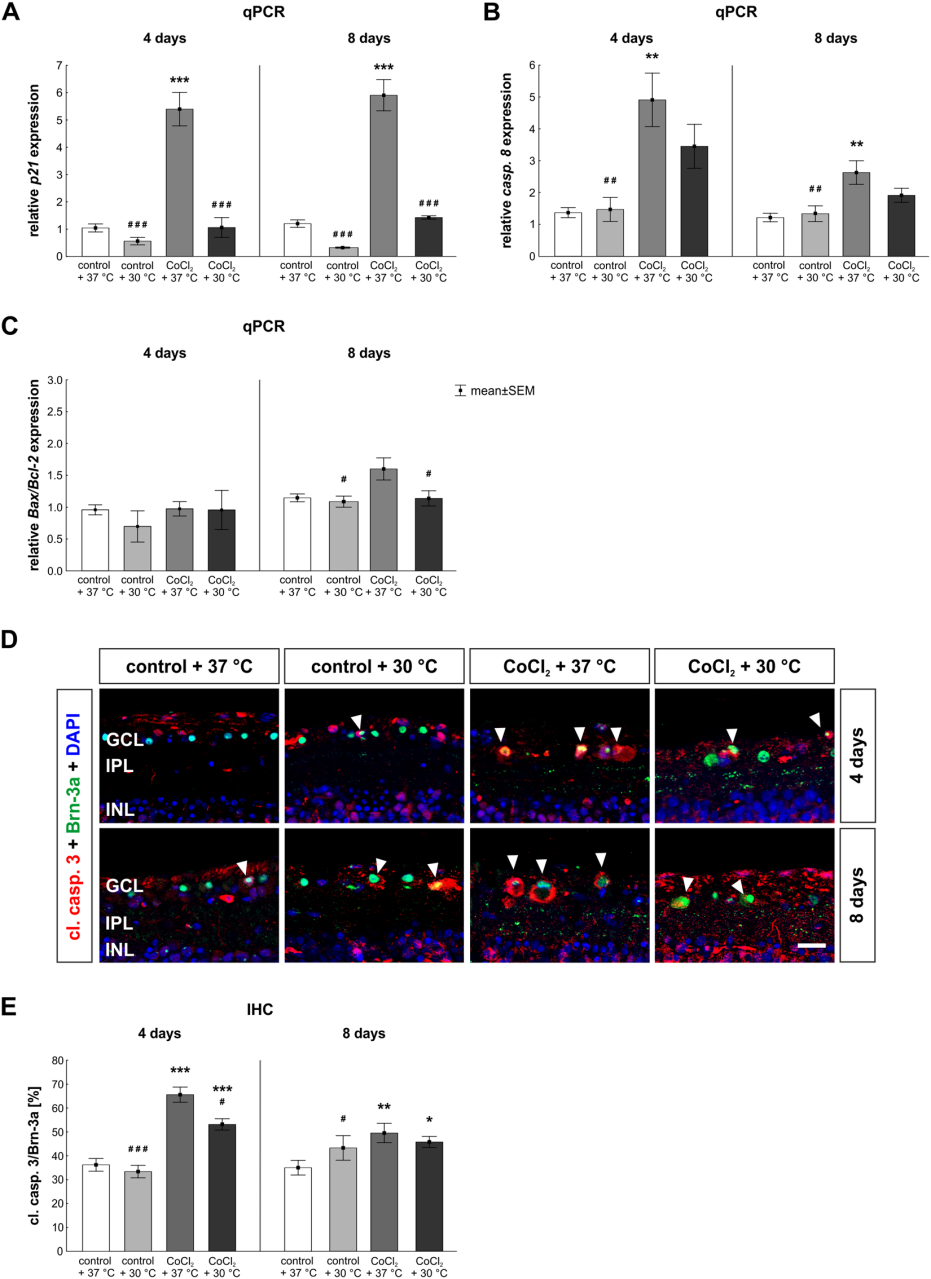
Inhibition of apoptotic processes via hypothermia. (**A**) Expression of the cell arrest gene *p21* was evaluated via qPCR. Analysis revealed that *p21* gene expression was strongly increased after four and eight days in the CoCl_2_ + 37 °C group. Hypothermia reduced the expression significantly at both points in time. (**B**) qPCR analyses of *caspase 8*. CoCl_2_ led to a strongly elevated expression of *caspase 8* in the CoCl_2_ + 37 °C group after four and eight days. Again, hypothermia treatment counteracted that effect. (**C**) Ratio of *Bax/Bcl-2* mRNA was measured via qPCR. After eight days, the *Bax/Bcl-2* ratio in the CoCl_2_ + 37 °C group tended to be increased. Hypothermia reduced the *Bax/Bcl-2* ratio in the CoCl_2_ + 30 °C group and no differences were seen in comparison to the control + 37 °C group. (**D**) Representative apoptotic retinal ganglion cells. RGCs were stained at four and eight days with anti-Brn-3a (RGCs; green) and cl. casp. 3 (apoptosis; red; arrowheads). Cell nuclei were visualized with DAPI (blue). (**E**) After four and eight days, the amount of apoptotic RGCs was significantly increased in the CoCl_2_ + 37 °C group. Interestingly, the number of apoptotic RGCs was reduced through hypothermia at four days. However, at eight days, no effects of hypothermia were detectable in the CoCl_2_ + 30 °C group compared to the CoCl_2_ + 37 °C. Abbreviation: GCL = ganglion cell layer; IPL = inner plexiform layer; INL = inner nuclear layer; qPCR = quantitative real-time PCR; IHC = immunohistochemistry. Values are mean ± SEM, A-C: n = 6–7/group; E: n = 10/group. Statistical differences to control + 37 °C group are marked with * and differences to CoCl_2_ + 37 °C group with #. ^#^,*p < 0.05; ^##^,**p < 0.01; ^###^,***p < 0.001. Scale bar = 20 µm.

The mRNA expression of *p21*, a well-known regulator of cell cycle arrest under stress conditions, was strongly elevated in the CoCl_2_ + 37 °C group (5.4 ± 1.5-fold; p = 0.0002; Fig. 6A). This effect was completely counteracted by hypothermia (CoCl_2_ + 30 °C: 1.1 ± 0.9-fold; p = 0.0002), leading to no differences between CoCl_2_ + 30 °C and control + 37 °C retinae (p > 0.9). The same effects were seen after eight days. A strong reduction of *p21* mRNA expression was noted in control + 30 °C retinae (0.3 ± 0.1-fold) compared to control + 37 °C ones (p = 0.181). Once again, the overexpression of *p21* mRNA in CoCl_2_ + 37 °C stressed retinae (5.9 ± 1.5-fold; p = 0.0002) was successfully reduced after hypothermia (CoCl_2_ + 30 °C: 1.4 ± 0.2-fold; p = 0.0002), even leading to no differences between the CoCl_2_ + 30 °C and control + 37 °C retinae (p > 0.9; Fig. 6A).

Apoptosis can be induced in two different ways^20^. The extrinsic pathway is activated when a ligand binds to a specific “death” receptor. This leads to the activation of caspases, like caspase 8. Therefore, the early extrinsic apoptosis pathway was evaluated via *caspase 8* (*casp. 8*) expression (Fig. 6B). At day four, CoCl_2_ + 37 °C stressed retinae (4.9 ± 2.1-fold; p = 0.002) showed a significant higher mRNA expression of caspase 8 than control retinae. This effect was lowered by hypothermia treatment (CoCl_2_ + 30 °C: 3.45 ± 1.69-fold; p = 0.314), but an mRNA increased expression was still observable in comparison to the control + 37 °C group (p = 0.084; Fig. 6B). After eight days, the *caspase 8* mRNA expression was significantly increased in the CoCl_2_ + 37 °C retinae (2.6 ± 1.0-fold expression; p = 0.004). Once again, a reduction of temperature prevented the *caspase 8* overexpression in the CoCl_2_ + 30 °C group (1.9 ± 0.6-fold; p = 0.229) in comparison to the CoCl_2_ + 37 °C group. Protective effects of hypothermia were seen once again, since no alterations were detectable comparing the expression in both CoCl_2_ stressed groups (p = 0.244; Fig. 6B).

The other way to induce apoptosis is the intrinsic pathway, in which stress signals lead to a secretion of cytochrome c from the mitochondria into the cytoplasm^20^. This alters the activation state of several pro- or anti-apoptotic proteins, which then leads to apoptosis. The intrinsic apoptosis pathway was analyzed via the *Bax/Bcl-2* ratio (Fig. 6C). After four days, no changes were noted within all groups (Fig. 6C). At day eight, no difference was observable between hypothermia treated CoCl_2_ + 30 °C retinae (1.14 ± 0.31-fold; p > 0.9) and control + 37 °C ones. In contrast, a significant reduction of the *Bax/Bcl-2* ratio, comparing CoCl_2_ + 30 °C and CoCl_2_ + 37 °C groups (p = 0.048), was observable (Fig. 6C).

Based on the fact, that we could show that hypothermia lowers apoptotic processes and protects RGCs, we were interested in the amount of apoptotic RGCs. Hence, we performed double immunolabeling of RGCs using Brn-3a and cleaved caspase 3 (Fig. 6D,E). After four days, both CoCl_2_ treated groups (37 °C: 65.6 ± 3.2% cl. casp. 3^+^ RGCs; p = 0.0002; 30 °C: 53.2 ± 2.4% cl. casp. 3^+^ RGCs; p = 0.0006) displayed a significantly increased number of apoptotic RGCs in comparison to the control + 37 °C (36.2 ± 2.6% cl. casp. 3^+^ RGCs). Nevertheless, hypothermia treatment significantly inhibited apoptotic processes in the CoCl_2_ + 30 °C group in comparison to the CoCl_2_ + 37 °C group (p = 0.013; Fig. 6E). After eight days, the addition of CoCl_2_ led to a significantly elevated apoptosis rate in the CoCl_2_ + 37 °C group (53.3 ± 3.7% cl. casp. 3^+^ RGCs; p = 0.002) in comparison to the control + 37 °C (34.4 ± 2.9% cl. casp. 3^+^ RGCs). At this point in time, hypothermia did not have any inhibiting effects on the apoptosis rate of RGCs, since hypothermia treated CoCl_2_ + 30 °C retinae (47.9 ± 2.9% cl. casp. 3^+^ RGCs; p = 0.034) showed a higher apoptosis rate than the control + 37 °C ones (Fig. 6E).

As our results show, hypothermia counteracted the cell cycle arrest of retinal cells triggered by CoCl_2_. Both, the expression of caspase 8 and the amount of apoptotic RGCs were strongly reduced after hypothermia treatment, which indicates that hypothermia lowered the apoptotic mechanisms.

### Rescue of microglia and macroglia

**Figure 7:**
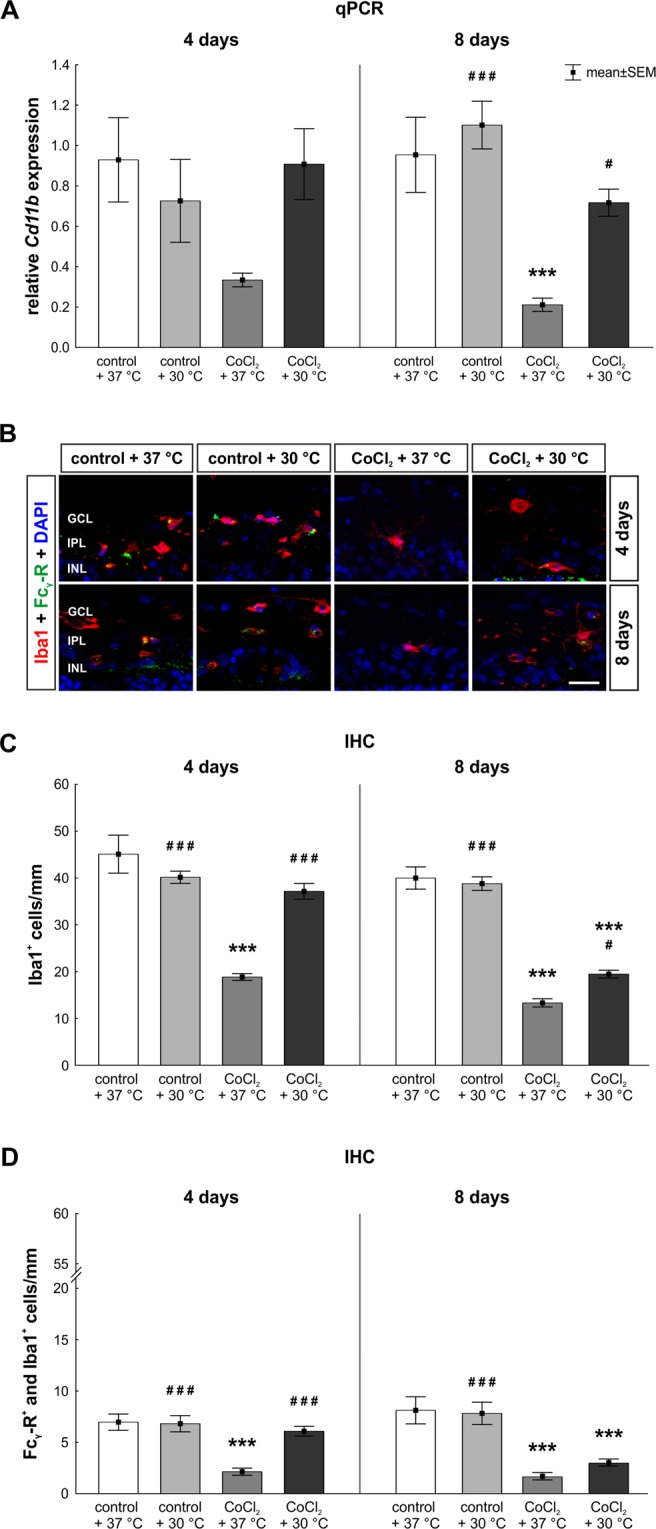
Hypothermia protected microglia. (**A**) Relative mRNA expression of *Cd11b*, a gene that is expressed by microglia, was analyzed via qPCR. At day four, a slightly decreased expression of *CD11b* was noted in the CoCl_2_ + 37 °C retinae, which was counteracted via hypothermia. After eight days, a significant reduction of *CD11b* was observed in the CoCl_2_ + 37 °C group. The damaging effect of CoCl_2_ was again counteracted by hypothermia in the CoCl_2_ + 30 °C group. (**B**) Representative pictures of microglia stained with anti-Iba1 (red) after four and eight days are shown. Anti-Fc_γ_-R was used as an activity marker for microglia (green). Fc_γ_-R^+^ and Iba1^+^ cells were counted as active microglia. Cell nuclei were visualized with DAPI (blue). (**C**) The significant loss of microglia due to CoCl_2_ was counteracted through hypothermia at both points in time. (**D**) CoCl_2_ led to a significantly reduced number of active microglia. Hypothermia treatment rescued active microglia at four days, but not at eight days. Abbreviations: GCL = ganglion cell layer; IPL = inner plexiform layer, INL = inner nuclear layer; qPCR = quantitative real-time PCR; IHC = immunohistochemistry. Values are mean ± SEM. A: n = 6–7/group; C, E: n = 10/group. Statistical differences to control + 37 °C group are marked with * and differences to CoCl_2_ + 37 °C group with #. ^#^p < 0.05; ^###^,***p < 0.001. Scale bar = 20 µm.

To evaluate microglia, *CD11b* mRNA expression, a gene which encodes for microglia receptors, was analyzed (Fig. 7A). A 3.3-fold reduction was noted in the CoCl_2_ + 37 °C group in comparison to the control + 37 °C group (p = 0.098) after four days. In accordance, protective effects of hypothermia were seen, since no alterations were observable between the control + 37 °C and CoCl_2_ + 30 °C group. After eight days, CoCl_2_ again induced a 5-fold reduction of *CD11b* mRNA expression in the CoCl_2_ + 37 °C group (p = 0.001). Hypothermia completely protected the *CD11b* mRNA expression in the CoCl_2_ + 30 °C group. Hence, no differences were notable compared to the control + 37 °C (p = 0.489; Fig. 7A).

To confirm previous findings, the total number of microglia was analyzed by Iba1 staining of retinal cross-sections (Fig. 7B). In accordance with the qPCR results, a loss of microglia was observable in the CoCl_2_ + 37 °C group after four days (18.8 ± 0.7 Iba1^+^ cells/mm; p = 0.0002) in contrast to the control + 37 °C group (45.1 ± 4.1 Iba1^+^ cells/mm). Hypothermia treatment counteracted the hypoxic effect of CoCl_2_, which resulted in a total rescue of microglia in the CoCl_2_ + 30 °C group (37.1 ± 1.7 Iba1 + cells/mm; p = 0.0002) compared to the CoCl_2_ + 37 °C group. Most importantly, no differences were found comparing the CoCl_2_ + 30 °C to the control group (Fig. 7C). After eight days, the number of Iba1^+^ cells was significantly lower in the CoCl_2_ + 37 °C group (13.3 ± 0.9 Iba1^+^ cells/mm; p = 0.0002). Again, hypothermia inhibited the impact of CoCl_2_ on microglia in the hypothermia treated CoCl_2_-stressed retinae (19.5 ± 0.8 Iba1^+^ cells/mm; p = 0.033). However, a loss of nearly 50% of the microglia population was still seen when comparing CoCl_2_ + 30 °C and control + 37 °C retinae (40.0 ± 2.4 Iba1^+^ cells/mm; p = 0.002; Fig. 7C).

In a next step, the number of active microglia was evaluated via Fc_γ_-R and Iba1 co-staining (Fig. 7B). A loss of active microglia was noted in the CoCl_2_ + 37 °C group (2.1 ± 0.3 Fc_γ_-R^+^ and Iba1^+^ cells/mm; p < 0.001) after four days. No differences between the hypothermia treated CoCl_2_-stressed retinae (CoCl_2_ + 30 °C: 6.1 ± 0.5 Fc_γ_-R^+^ and Iba1^+^ cells/mm; p = 0.774) and the control retinae (7.0 ± 0.8 Fc_γ_-R^+^ and Iba1^+^ cells/mm) proved a total rescue of active microglia through hypothermia (Fig. 7D). At day eight, the addition of CoCl_2_ induced a significant reduction of active microglia, irrespectively of the temperature (37 °C: 1.6 ± 0.4 Fc_γ_-R^+^ and Iba1^+^ cells/mm; p = 0.001; 30 °C: 3.0 ± 0.3 Fc_γ_-R^+^ and Iba1^+^ cells/mm; p = 0.003; control + 37 °C: 8.1 ± 1.3 Fc_γ_-R^+^ and Iba1^+^ cells/mm; Fig. 7D).

Investigations of macroglial response revealed a significantly reduced *GFAP* mRNA expression in both CoCl_2_ groups at day 4 (37 °C: 0.19 ± 0.20-fold p = 0.001; 30 °C: 0.40 ± 0.30-fold; p = 0.039; Sup. Fig. S2A). At day eight, no differences were observed within all investigated groups (Sup. Fig. S2A). Western blot analyses showed no changes in GFAP signal intensities in any of the groups neither at four nor at eight days (Sup. Fig. S2B,C). The evaluation of GFAP immunoreactivity revealed the same results as seen in qPCR analyzes (Sup. Fig. S2D). Significant lower GFAP signals were seen in the CoCl_2_ + 37 °C groups in both points in time (4 days: 8.30 ± 0.69 [%]/area; p = 0.029; 8 days: 13.99 ± [%]/area; p = 0.030) compared to the control retinae (4 days: 11.80 ± 0.41 [%]/area; 8 days: 20.12 ± 1.51 [%]/area; CoCl_2_ + 37 °C). In accordance with the results regarding microglia, hypothermia reduced the effect of CoCl_2_ also on macroglia. Only a slight reduction was noted comparing the control + 37 °C and the CoCl_2_ + 30 °C groups (4 days: 9.46 ± 0.34 [%]/area; p = 0.22; 8 days: 16.89 ± 0.94 [%]/area; p = 0.31; Sup. Fig. S2E).

## Discussion

The aim of this study was to investigate possible neuroprotective effects of hypothermia (30 °C) on CoCl_2_-stressed cultured porcine retina explants. We demonstrated that hypoxic damage, such as oxidative stress, due to CoCl_2_ on retinal cells was diminished through hypothermia. Furthermore, hypothermia had inhibiting effects on the apoptosis and led to an enhanced cell survival.

It has been described that cobalt, like hypoxia, triggers the stabilization of the α-subunit of hypoxia-inducible factor (HIF-1) by preventing its degradation^21^. Increased levels of HIF-1α activate the expression of certain genes, like *iNOS* and heat shock proteins (HSPs)^22,23^. Furthermore, cobalt leads to DNA fragmentation, caspase activation, and to ROS-production through the uncoupling of mitochondrial respiration^24,25^. Toxic effects of cobalt include a loss of mitochondrial membrane potential, the inhibition of the proteasome degradation, resulting in cell death^22,26^. Nevertheless, the treatment of CoCl_2_ can simulate a disease process and can cause symptoms very similar to those of hypoxia^22^.

Due to the fact, that CoCl_2_ stabilizes HIF-1α, *HIF-1α* mRNA expression was investigated to verify that hypoxia was successfully induced in retinae stressed with CoCl_2_. In our study, CoCl_2_-stressed hypoxic retinae presented a higher *HIF-1α* mRNA expression as well as a higher number of hypoxic cells, showing that CoCl_2_ successfully induced hypoxia. The stabilization of HIF-1α results in a higher expression of genes that encode several proteins, like heat shock proteins (HSPs) and inducible nitric oxide synthase (iNOS), which are essential to manage hypoxic stress^27^. This effect was successfully seen in CoCl_2_-stressed retinae, in which the mRNA expression of HSP70 was strongly elevated^22^. HSPs are chaperons which are important for the defense against cellular stress. They prevent misfolding and protein aggregations. Especially HSP70 is required for the transcriptional activity as well as for the accumulation and function of HIF-1α^28^. Our results show that hypothermia led to a strong reduction not only of HIF-1α but also of HSP70, pointing out that HIF-1α and HSP70 are strongly linked. The same indirect effect of CoCl_2_ on HSP70 was also described in other studies, strengthening our suggestions^29,30^. Nevertheless, our results do not clarify, whether hypothermia first inhibits HIF-1α accumulation which than leads to the reduction of HSP70, or whether hypothermia prevents the HSP70 expression which results in a reduced HIF-1α amount.

Divalent metal ions, such as cobalt, induce a disturbance of the mitochondrial respiration chain and stimulate the rupture of the outer cell membrane, resulting in ROS production and oxidative stress^6,8,22^. Our results suggest that CoCl_2_ not only mimics hypoxia through the stabilization of HIF-1α, but also leads to a strongly elevated level of ROS, indicating that it triggers oxidative stress. In accordance with other publications, our results show that hypoxia and oxidative stress trigger apoptotic mechanisms^24,25^, which then led to the significant loss of RGCs in the retinae. These mechanisms were completely counteracted by hypothermia, which probably first blocked the interaction of CoCl_2_ and HIF-1α, then HSP70 and iNOS were strongly reduced, leading to decreased oxidative stress and alleviated apoptosis mechanisms.

The prominent loss of RGCs was associated with an increased *p21* mRNA expression, a gene which induces cell cycle arrest, and *caspase 8*, a hallmark for extrinsic apoptosis^20^. An overexpression of *HIF-1α* can induce apoptotic processes in different ways: the interaction of HIF-1α and p53, a tumor suppressor gene, leads to the activation of apoptotic mechanisms^2^. In addition, HIF-1α activates p21 and lowers the cell viability^31^.

As mentioned before, a *HIF-1α* overexpression was observed in CoCl_2_-stressed retinae at both points in time. In the early point in time, hypothermia totally stabilized the expression of HIF-1α to control level. This effect was accompanied by a total reduction of cellular stress, namely control-like HSP70 and iNOS expression- as well as ROS-levels, causing strongly lowered extrinsic apoptosis. After eight days, qPCR analyzes regarding HIF-1α indicate that the effect of hypothermia was lower than after four days. Anyway, the reduced number of HIF-1α^+^ cells in the retina show that after eight days there was still a positive effect through hypothermia.

In accordance, after eight days, *p21* expression was diminished in the hypothermia treated CoCl_2_-stressed retinae and *caspase 8* expression was lower than in CoCl_2_ retinae. Hence, hypothermia led to an early inhibition of hypoxic processes and reduced the apoptosis. In addition, hypothermia inhibited caspase 3 at the earlier, but not at the later point in time. It is known that hypothermia protects cells through a diminished apoptosis rate by inhibiting the lactate dehydrogenase^32^ and by reducing the caspase 3 expression^33^. Moreover, it was shown for retinal explants of mice, that hypothermia treatment during the retinal dissection led to a strongly decreased apoptosis rate in the retina^34^. Caspase 8 is activated at an early stage of extrinsic apoptosis, whereas caspase 3 is cleaved in later stages of intrinsic apoptosis^35^. A possible explanation would be that hypothermia rather inhibits the extrinsic than the intrinsic pathway. Furthermore, other upstream proteins, besides caspase 8, might be the reason for the lacking inhibition of caspase 3 after hypothermia at the later point in time.

Bax is a pro-apoptotic protein, which induces the intrinsic apoptosis by opening mitochondrial pores and supporting the secretion of cytochrome c into the cytoplasm. Bcl-2, on the other hand, is an anti-apoptotic protein that inhibits Bax and prevents the secretion of cytochrome c^36^. In our study, the *Bax/Bcl-2* ratio was slightly increased in CoCl_2_-stressed retinae after eight days, but not in hypothermia treated CoCl_2_-stressed ones. Hence, hypothermia seems to inhibit the apoptosis via increasing the *Bcl-2* expression or decreasing the *Bax* expression. Several studies revealed that CoCl_2_-induced hypoxia leads to apoptosis. Kuehn *et al*. demonstrated that CoCl_2_-treated porcine retinae showed an increased *Bax* expression level after four days of cultivation^11^. Chang *et al*. observed that hypoxia activates the intrinsic apoptosis via Bax and caspase 3, whereas Lee *et al*. reported that CoCl_2_ leads to apoptosis by activating both pathways simultaneously^37,38^.

In previous studies a loss of calretinin^+^ amacrine and PKCα^+^ bipolar cells was noted through CoCl_2_^6,11^. This is the first study that shows a time dependent damage due to CoCl_2_ on calretinin^+^ amacrine cells, parvalbumin (*PVALB*) expressing displaced amacrine as well as PKCα^+^ bipolar cells.

Amacrine cells are located in the inner nuclear layer (INL) and play an important role for the modulation of signals to the RGCs^39^. It is known that they are vulnerable to glaucomatous damage^40–42^. There is a strong connection between amacrine cells and RGCs via gap-junctions, which leads to a secondary loss of amacrine cells after glaucoma-induced RGC loss^42^. In good accordance, we observed a delayed loss of amacrine cells at eight days which was induced by CoCl_2_ and possibly strengthened by the early loss of RGCs. Hypothermia did not protect amacrine cells. We assume that the RGC loss due to CoCl_2_ led to severe changes in the surrounding tissue, where dendrites of amacrine cells are located and therefore the protection of amacrine cells cannot be achieved.

Bipolar cells are also located in the INL and are connected to rods or cones. We used PKCα to label rod bipolar cells. A time-dependent loss of rod bipolar cells was observed. This was counteracted by hypothermia treatment. This later death is in accordance with affected bipolar cells in different rat glaucoma models^41,43^. Since hypothermia had a rescue effect on bipolar cells, but not on amacrine cells, we assume that the degeneration process of both cell types is different. To confirm this, further studies are necessary.

Besides HSP70, also the expression of inducible nitric oxide synthase (iNOS) depends on the activity of HIF-1α. CoCl_2_ has degenerative effects on microglia through increasing the apoptosis rate and diminishing the cell viability via cell arrest^11,44^. Besides neurons, microglia were also protected by hypothermia. Interestingly, the gene encoding for the enzyme iNOS, which is mainly produced by microglia, was increased by hypoxic stress without a microglia response. HIF-1α might be involved in this pathway, since it is known that HIF-1α increases the *iNOS* expression by binding the transcription promotor^45,46^. Therefore, in our model iNOS regulation seems to be independent from a microglia response.

*GFAP* expression, is a hallmark for gliosis in retinal diseases or injuries. Macroglial signals in the porcine retina are stronger than in rodent retinae^47,48^. However, we detected a reduced *GFAP* expression in qPCR and immunohistochemical analyses, while western blot analyses revealed no changes in GFAP signal intensities at any of the investigated points in time. Those results indicate that CoCl_2_ seems to have toxic effects not only on microglia but also on macroglia. However, our study and other studies reveal that in porcine degeneration models gliosis is not occurring during cultivation^48–50^. The preparation of retinal explants seems to induce a macroglial response itself. Therefore, it is difficult to detect further changes. Furthermore, in our *ex vivo* model, retinae are cultivated separate from the optic nerve. Thus, the immigration of astrocytes as a result of macrogliosis is not possible.

## Conclusion

Hypoxic processes play a crucial role in several retinal diseases. Cobalt chloride (CoCl_2_) is known to mimic hypoxic processes *in vitro* by stabilizing the transcription factor HIF-1α and leading to oxidative stress through a disruption of mitochondrial respiration. These effects were observed in the present study. This hypoxic damage due to CoCl_2_ was associated with oxidative stress leading to increased apoptosis rates in CoCl_2_-stressed retinae. We demonstrated that hypothermia completely counteracted these mechanisms by probably disturbing the interaction of CoCl_2_ and HIF-1α. This led to strongly reduced HSP70 and iNOS synthesis, alleviating oxidative stress and preventing apoptosis. Consequently, most RGCs and bipolar cells were rescued, while amacrine and horizontal cells were not protected.

In conclusion, we demonstrated that our CoCl_2_-induced hypoxia is a suitable model system to test potential therapies and that hypothermia could be a possible additional treatment for retinal diseases.

## Methods

### Preparation of retinal explants

Porcine eyes were obtained from the local abattoir and retinae were prepared within three hours from enucleation. The preparation of retinal explants was performed as described previously^11,49,51^. Briefly, the eyeball was opened to separate anterior parts of the eye from the eye cup. The eye cup was incised four times to produce a cloverleaf-like shape. Next, one retinal explant sample per leaf was punched out in the central part of the retinal quadrant using a dermal punch (Ø = 6 mm, Pfm medical AG). Remaining retinal pigment epithelium was removed by washing retinal explants in Neurobasal-A medium (Life Technologies). Finally, retinal samples were placed on a Millicell culture insert (Millipore) with the GCL facing up and cultured in Neurobasal-A medium (Life Technologies) supplemented with 0.8 mM L-glutamine (Life Technologies), 2% B27 (Life Technologies), 1% N2 (Life Technologies) and 2% penicillin/streptomycin (Sigma-Aldrich), for four and eight days. Medium was exchanged completely at days zero, one, two and three. Additionally, half of the medium volume was replaced at days five and seven (Fig. 1A). The substance most commonly used to simulate a hypoxic environment is CoCl_2_^8,9,52,53^, hence this was used in the current study. Hypothermia treatment and hypoxia induction via 300 µM CoCl_2_ (Sigma-Aldrich) were performed simultaneously and took 48 h in total (Fig. 1A). Control groups were cultivated without the stressor and with or without additional hypothermia treatment, so that four groups were compared: control + 37 °C, control + 30 °C, CoCl_2_ + 37 °C and CoCl_2_ + 30 °C.

At days four and eight, retinal explants were obtained for quantitative real-time-PCR (qPCR, n = 6–7/group/point in time), histological (n = 9–10/group/point in time) and western blot analyses (n = 4/group/point in time; Fig. 1A).

### Measurement of reactive oxygen species (ROS)-level and pH-value

For the measurement of ROS-level in cultured retinae, the non-lytic protocol of ROS-GLO™ H_2_O_2_ assay (Promega) was performed. Retinae were cultured as described before and the measurement of ROS-level was performed according to the manufacture’s protocol. For more detailed description please see supplementary part. The measurement of the pH-values was performed with LAQUAtwin B-712. Calibration was performed according to manufactural instructions. After the medium exchange, 200 µl medium was used for the measurement.

### Quantitative real-time PCR (qPCR)

The used primer for qPCR analyses are given in Supplementary Table 1. qPCR analyses were performed as described previously^11,49,51^ for n = 6/group at day four and n = 7/group at days four and eight. All target genes were normalized against housekeeping genes encoding *Histone H3* and *β-Actin* (Sup. Table 1). C_t_-Values of both housekeeping genes were not affected. The mean of all samples for β-actin was 18,84 ± 0.8 cycles and for histone H3 20.22 ± 0.9 cycles. Samples having C_t_-values 2 cycles higher or lower than the mean were excluded. Geometric mean of the C_t_-Values of both genes were calculated and used as reference. For the relative quantification the Δ-ΔC_t_- algorithm, with efficiency corrected calculation model, based on one sample was used^54^. All groups were compared to control + 37 °C or CoCl_2_ + 37 °C groups.

### Histological analysis of retinal cells

For immunohistochemical analyses retinal cross-sections (n = 9–10/group/point in time) and flatmounts of retinae (n = 3/group) were prepared as described previously^11,49,51^. To identify different cell types and proteins specific primary antibodies and matched secondary antibodies were used (Sup. Table 2). For all stainings, 4′,6 diamidino-2-phenylindole (DAPI) was used to visualize the cell nuclei. Cross-sections and flatmounts were blocked with blocking-buffer containing 10–20% donkey or goat serum and 0.1–0.2% TritonX in PBS. Six cross-sections were stained per retina and cells were counted in 24 masked and defined image sections using ImageJ software. Regarding the evaluation of flatmounts, 9 images, including 4 peripheral and 5 central parts of the retina, were counted. For GFAP, the area was measured using an established protocol and an ImageJ macro^40,41^. For more information please see supplementary part.

### Western blot

Western blot analyses were performed (n = 4/group/point in time) as described previously^11,49,51^. To this end, the primary antibodies (Sup. Table 2), diluted in the blocking solution, were incubated over night at 4 °C. After the washing steps, the secondary antibodies (Sup. Table 2) were applied for 60 min. Protein bands were recorded at 700 and 800 nm and evaluated with the Odyssey infrared imager system 2.1 (LI-COR Bioscience). HSP70, (70 kDa), β-III-tubulin (55 kDa) and GFAP (55 kDa) signal intensities were normalized to β-actin (42 kDa) signal intensities.

### Statistical analyses

Groups were compared by one-way ANOVA, followed by Dunnett’s post-hoc test (Statistica V 12; Statsoft). Results are presented as mean ± SEM. A p-value < 0.05 was considered as statistically significant. The level of significance was set to *p < 0.05, **p < 0.01, ***p < 0.001. Statistical differences compared to the control + 37 °C group are shown with *, differences compared to the CoCl_2_ + 37 °C group are shown with^#^.

## Supplementary information

Supplementary information
