# Supplementary material for: Diminished apoptosis in hypoxic porcine retina explant cultures through hypothermia
Source: Sci Rep. 2019 Mar 20;9:4898. doi: 10.1038/s41598-019-41113-4 (PMC6427006; doi:10.1038/s41598-019-41113-4)
Supplement: Supplementary file 1 — Supplementary information [file 41598_2019_41113_MOESM1_ESM.pdf]

Diminished apoptosis in hypoxic porcine retina explant cultures through hypothermia

Ana M. Maliha[1], Sandra Kuehn[1], José Hurst[2], Fenja Herms[1,3], Michael Fehr[3], Karl U. Bartz-Schmidt[2], H. Burkhard Dick[1], Stephanie C. Joachim[1#], Sven Schnichels[2#]

1: Experimental Eye Research Institute, University Eye Hospital, Ruhr-University Bochum, Bochum, Germany

2: University Eye Hospital Tübingen, Centre for Ophthalmology Tübingen, Tübingen, Germany

3: Clinic for Small Animals, University of Veterinary Medicine, Hannover, Germany

#: equal contributing authors and corresponding authors:

Dr. Sven Schnichels

University Eye Hospital Tübingen, Centre for Ophthalmology, Elfriede-Aulhorn-Str. 7, 72076 Tübingen, Germany

Phone: +49-7071-2987888, Fax: +49-7071-294935

E-Mail: sven.schnichels@med.uni-tuebingen.de

PD Dr. Stephanie C. Joachim

Experimental Eye Research, University Eye Hospital, Ruhr-University Bochum, In der Schornau 23-25, 44892 Bochum, Germany

Phone: +49-234-299-3156, Fax: +49-234-299-3157

E-Mail: stephanie.joachim@rub.de

Supplementary Figure S1: Hypothermia protected retinal thickness. **A)** Retinal cross-sections were stained with hematoxylin & eosin to evaluate retinal thickness. **B)** $CoCl_2$ led to a strong reduction of retinal thickness after four and eight days. Hypothermia prevented the reduction due to $CoCl_2$. Hypothermia treated $CoCl_2$-stressed retinae had a similar thickness to control retinae. Abbreviations: GCL = ganglion cell layer; IPL = inner plexiform layer; INL = inner nuclear layer; OPL = outer plexiform layer; ONL = outer nuclear layer. n=8-10/group. Statistical differences to control + 37 °C group are marked with * and differences to $CoCl_2$ + 37 °C group with #. *: p<0.05; $^{\#\#}$, **: p<0.01. Scale bar=20µm.

Supplementary Figure S2: Rescue of macroglial response through hypothermia. **A)** mRNA expression of *GFAP* was significantly reduced in both $CoCl_2$ treated groups compared to the control + 37 °C group at four days as observed in qPCR analyses. After eight days, the *GFAP* expression was not affected in any of the groups compared to the control + 37 °C group. A slightly increased expression was noted between the $CoCl_2$ + 30 °C and the $CoCl_2$ + 37 °C group. **B)** Protein levels of GFAP (55 kDa) were normalized against β-III-tubulin (42 kDa) and evaluated via western blot analyses. **C)** Western blot analyses revealed comparable GFAP protein levels in both points in time. **D)** $GFAP^+$ area was measured in retinal cross-sections. **E)** Treatment with $CoCl_2$ led to a significantly reduced $GFAP^+$ area. Hypothermia counteracted the harmful effect of $CoCl_2$ due to macroglia and normalized the $GFAP^+$ area in $CoCl_2$ + 30 °C treated retinae. Abbreviations: GCL = ganglion cell layer; IPL = inner plexiform layer; INL = inner nuclear layer; OPL = outer plexiform layer; ONL = outer nuclear layer. Values are mean±SEM, A: n=6-7/group; C: n=4/group; E: n=10/group. Statistical differences to

control + 37 °C group are marked with * and differences to $CoCl_2$ + 37 °C group with #. $^{\#}$, *: $p<0.05$; **$p<0.01$. Scale bar=20µm.

Supplementary Table 1: qPCR primer pairs. The listed primer pairs were used in quantitative real-time PCR experiments.

Supplementary Table 2: Listed primary and matched secondary antibodies were used for immunohistological analyses.

Supplementary Figure S1:

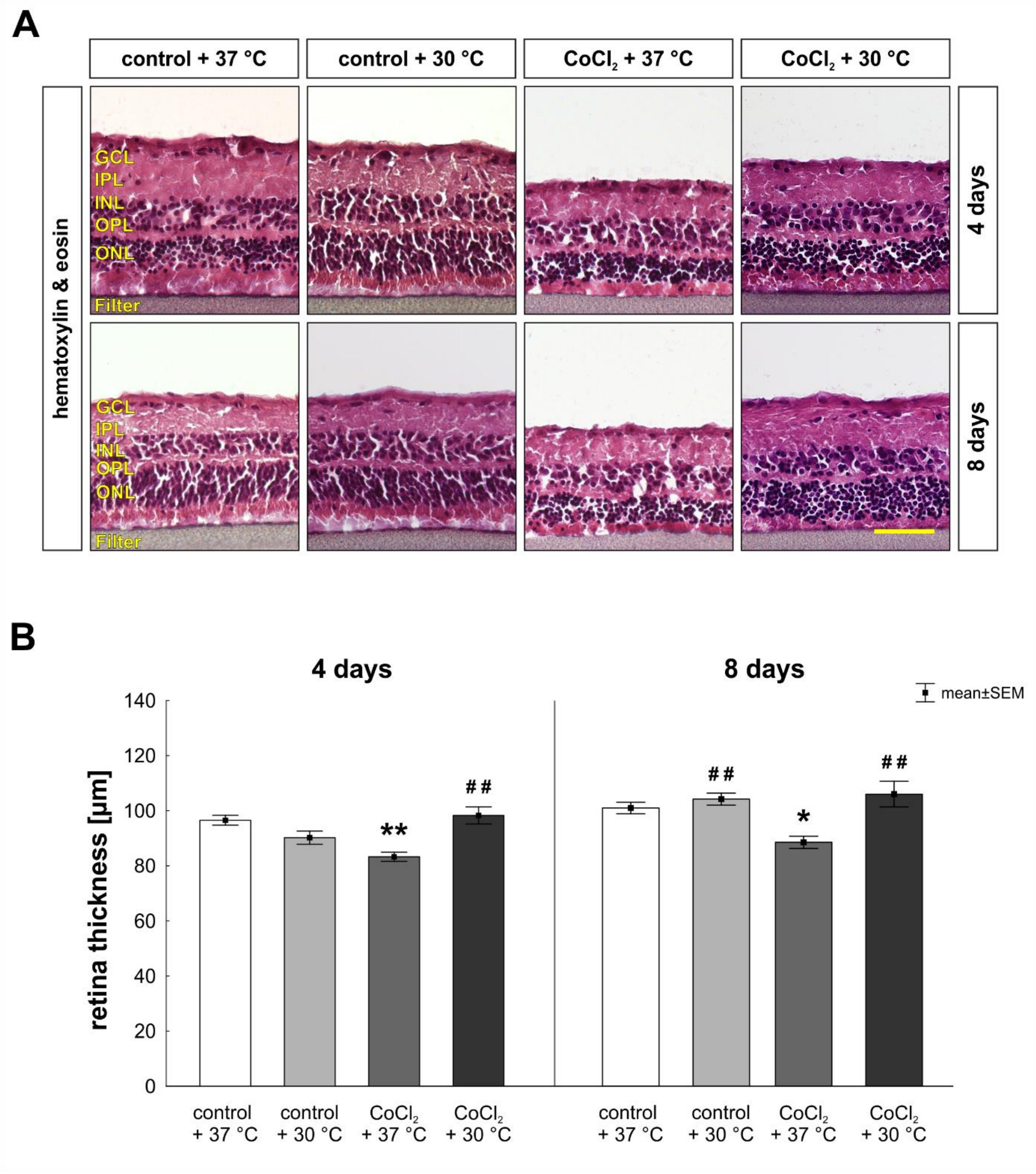

Supplementary Figure S2:

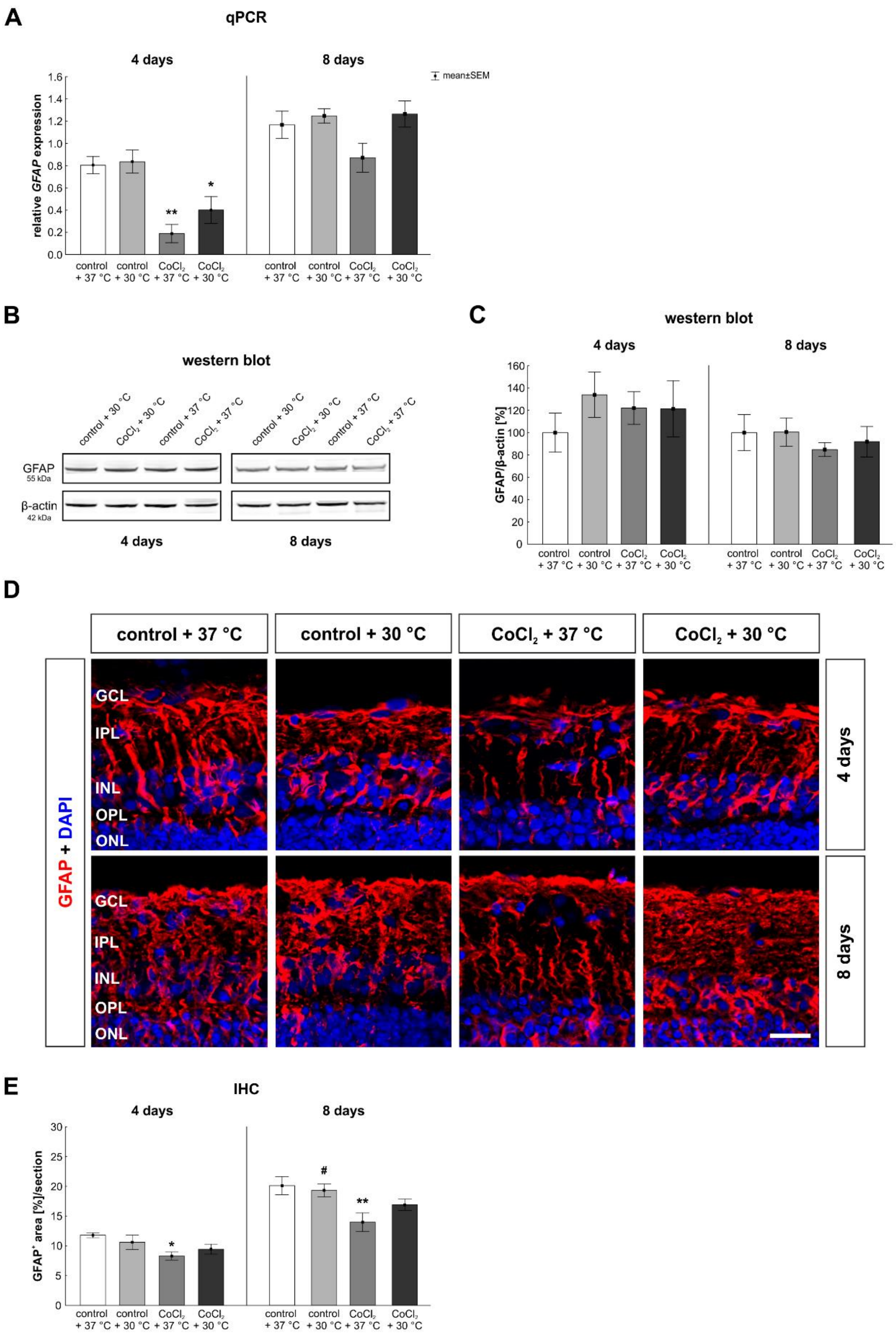

Supplementary Table 1:

Gene	Oligonucleotide sequence
Bax	5’GGACCATCGGTATTGGTGTC3’<br>3’AGATGAGGGAGAGAGGCACA5’
Bcl-2	5’GCTCGTGCGGGATTGACTACTACA3’<br>3’CCAGCGGGTTCTTGCCACAGC5’
Calbindin	5’TGAACCCAAGCTCCAAGAGT3‘<br>3’AAAAGGTGAAGATGGCGTTG5’
Caspase 8	5’GCCCAGATCTCTGCCTACAG3’<br>3’CAGGGCCTTGTTGATTTGTT5’
GFAP	5’CAGGATCTGCTCAACGTCAA3’<br>3’ATCTCCACGGTCTTCACCAC5’
Histon H3	5’ACTGGCTACAAAAGCCGCTC3’<br>3’ACTTGCCTCCTGCAAAGCAC5’
HSP70	5’ATGTCCGCTGCAAGAGAAGT3’<br>3’GGCGTCAAACACGGTATTCT5’
PVALB	5’CAACGCTGAGGACATCAAGA3’<br>3’TGACAGGTCTCTGGCATCTG5’
TUBB3	5’CAGATGTTCGATGCCAAGAA3’<br>3’GGGATCCACTCCACGAAGTA5’
β-actin	5’CTCTTCCAGCCTTCCTTC3’<br>3’GGGCAGTGATCTCTTTCT5’
CD11b	5’AGAAGGAGACACCCAGAGCA3’<br>3’GTAGGACAATGGGCGTCACT5’
iNOS	5’TGTTCAGCTGTGCCTTCAAC3’<br>3’CAGAACTGGGGGTACATGCT5’
p21	5’GACCCTCAGAAGAGCCACAG3’<br>3’GTCGAAGTTCCATCGCTCTC5’
Calbindin	5’TGAACCCAAGCTCCAAGAGT3’<br>3’AAAAGGTGAAGATGGCGTTG5’
HIF1α	5’TTACAGCAGCCAGATGATCG3’<br>3’TGGTCAGCTGTGGTAATCCA5’

Supplementary Table 2:

	Primary antibodies				Secondary antibodies			
	**Antibody**	**Company**	**Catalogue#**	**Dilution**	**Antibody**	**Company**	**Catalogue#**	**Dilution**
**IHC**	Anti-Brn-3a	Santa Cruz	SC-31984	1:100	Donkey anti-goat Alexa Fluor 488	Dianova	705-545-147	1:500
	Anti-calretinin	Millipore	AB1550	1:2000				
	Anti-Chx10	Santa Cruz	SC-21692	1:300				
	Anti-cleaved caspase 3	Sigma-Aldrich	C8487	1:300	Donkey anti-rabbit Alexa Fluor 555	Jackson Immuno-Research	711-547-003	1:500
	Anti-Fcγ-R (CD16/32)	BD Bioscience	14-0161	1:100	Donkey anti-rat Alexa Fluor 488	Thermo Fischer	A-21208	1:400
	Anti-GFAP	Millipore	AB5541	1:3000	Donkey anti-chicken Cy3	Millipore	AP194C	1:500
	Anti-HIF-1α	BD Bioscience	610959	1:100	Donkey anti-mouse Alexa Fluor 555	Abcam	AB150106	1:500
	Anti-Iba1	WAKO	019-19741	1:400	Donkey anti-rabbit Alexa Fluor 555	Jackson Immuno-Research	711-547-003	1:500
	Anti-NeuN	Millipore	ABN91	1:400	Donkey anti-chicken Alexa Fluor 488	Jackson Immuno-Research	703-545-155	1:500
	Anti-PKCα	Santa Cruz	SC-8393	1:300	Donkey anti-mouse Alexa Fluor 555	Abcam	AB150106	1:500
**WB**	Anti-GFAP	Millipore	AB5541	1:3000	IRDye donkey anti-chicken Alexa Fluor 680	LI-COR	925-68075	1:20000
	Anti-HSP70	Santa Cruz	SC-24	1:200	Donkey anti-mouse Alexa Fluor 680	Invitrogen	A10038	1:5000
	Anti-β-III-tubulin	R&D Systems	MAB1195	1:10000				
	Anti-β-actin	Cell Signaling	4970S	1:5000	IRDye donkey anti-rabbit DL800	Thermo Fischer	SA5-10044	1:20000
	Anti-β-actin	Sigma-Aldrich	S2228	1:6000	IRDye donkey anti-mouse DL800	LI-COR	926-32212	1:20000

Supplementary text

Methods

Measurement of reactive oxygen species (ROS)-level

On day one, right before $CoCl_2$-treatment, part of the filter inserts with the retinae were cut out, placed in a 12-well plate and cultivated in 500 µl media containing 300 µM $CoCl_2$. The next day, 18 h after $CoCl_2$-treatment started, H_2O_2 substrate was added to the medium and placed in the incubator for another 6 h. Then, 50 µl of a ROS-GLO™ Detection Solution was added to a 96-well plate (Greiner). To detect ROS in cultured retinae, 50 µl media of each retinae was added to the 96-well plate and incubated for 20 minutes at room temperature. ROS-level was measured with GloMax® 96 Microplate Luminometer (Promega) with an exposure time of 0.5 seconds. For the measurement 48 hours after $CoCl_2$-induction, the same protocol was performed on day three of the cultivation.

Histological analyses of retinal cells

For GFAP measurement, all images were cropped (600x700 pixel). Next, images were transformed into grayscale and for each image, a suitable upper and lower threshold was evaluated. The mean value of all upper and lower thresholds was calculated for the respective point in time and used for the final analyses (4 days: lower threshold: 10.42; upper threshold: 84.41; 8 days: lower threshold: 13.95; upper threshold: 85.05).
